# Severe thrombocytopenia due to idiopathic myelodysplastic syndrome complicated by spontaneous, fatal intracerebral hemorrhage

**DOI:** 10.1016/j.clinsp.2023.100227

**Published:** 2023-06-16

**Authors:** Carla Alexandra Scorza, Antonio-Carlos G. de Almeida, Fulvio Alexandre Scorza, Josef Finsterer

**Affiliations:** a Disciplina de Neurociência, Universidade Federal de São Paulo/Escola Paulista de Medicina (UNIFESP/EPM), São Paulo, SP, Brazil; b Centro de Neurociências e Saúde da Mulher “Professor Geraldo Rodrigues de Lima”, Escola Paulista de Medicina, Universidade Federal de São Paulo (EPM/UNIFESP), São Paulo, SP, Brazil; c Neurology & Neurophysiology Center, Vienna, Austria

Myelodysplastic Syndromes (MDSs) are a group of disorders that occur predominantly in men over the age of 65 years and are characterized by poorly formed or malfunctioning blood cells.^1^^,^^2^ The cause of MDSs often remains elusive, but some patients have a history of radiotherapy or chemotherapy. Common measures include blood transfusions and drugs to increase blood cell production. In certain situations, a Bone Marrow Transplant (BMT), also known as a stem cell transplant, may be recommended to replace the bone marrow with healthy bone marrow from a donor.^1^^,^^2^ MDSs are often complicated by the development of malignancy, infections, stroke, or bleedings due to thrombocytopenia.^1^^,^^2^ Although Intracerebral Bleeding (ICH) has been reported in association with MDS^3^, spontaneous ICH due to severe thrombocytopenia secondary to MDS has not been reported.

An 81-year-old Caucasian male, height 160 cm, weight 58 kg, was diagnosed with MDS Refractory Anemia with Excessive Blasts (RAEB) II on a bone marrow biopsy performed to work up a previously unknown pancytopenia (Table 1). A cause for MDS could not be identified. His history was positive for left middle cerebral artery ischemic stroke 30y previously with residual mild, right-sided, spastic hemiparesis and sensorimotor aphasia, well-controlled arterial hypertension, benign prostatic hyperplasia, ileus 10y earlier, and bilateral pneumonia. During two hospital stays due to constipation or pneumonia shortly before the ICH, he was treated with platelet concentrates and erythrocyte concentrates (Table 1). Severe thrombocytopenia recurred a few days after each transfusion.

**Table 1:** Results of blood tests shortly before the fatal bleeding.

Parameter	RL	dbd79	dbd74	dbd71^a^	dbd32	dbd29	dbd27^a^	dbd25^a^	dbd4
Erythrocytes	4.2-5-5/pL	2.8	2.53	3.46	2.8	1.87	2.65	2.79	2.11
Hemoglobin	14‒17g/dL	8.7	8.1	10.8	8.8	5.6	8.0	8.8	6.3
Hematocrit	40‒50%	25.2	22.8	30.6	24.4	16.8	23.4	24.2	22.1
Thrombocytes	150‒450 nL	27	33	53	11	5	36	51	11
Reticulocytes	5‒15%	18	Nd	nd	nd	nd	nd	nd	nd
Leucocytes	4‒9 nL	2.2	1.8	2.3	2.8	1.6	2.0	1.8	1.5
PTZ	70‒130%	61	65	nd	66	nd	66	nd	nd
PTT	<34s	21	29	nd	27.7	nd	27.4	nd	nd

dbd, Days before decease; nd, Not done; PTT, Partial Thromboplastin Time; PTZ, Prothrombin Time; RL, Reference Limits.

a fter thrombocyte concentrates and erythrocyte concentrates.

Two months after diagnosis of MDS, the patient suddenly became comatose with non-reactive pupils. His blood pressure was 145/77 mmHg. Platelet count was 11 nL four days before ICH (Table 1). emergency Cerebral Computed Tomography (CCT) revealed a left, frontotemporal, intracerebral mass bleeding with ventricular rupture (Fig. 1). Neurosurgeons saw no indication for surgical rehabilitation of the bleeding.

**Fig. 1:**
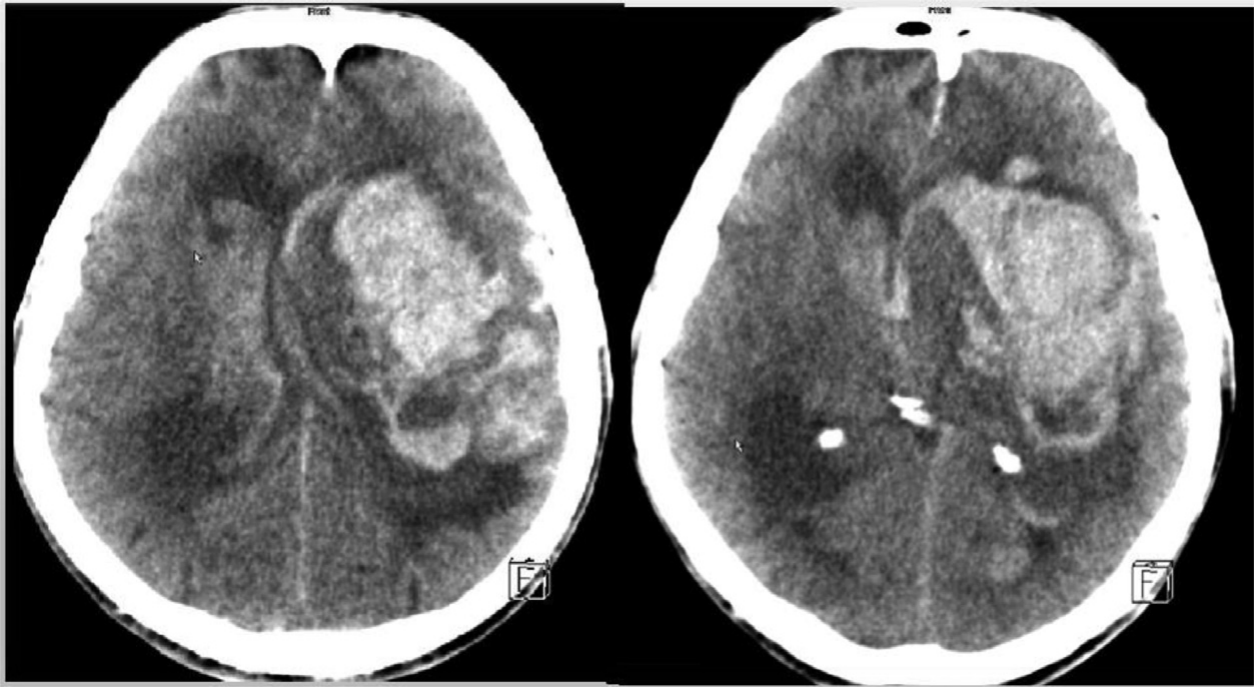
Cerebral CT scan showing intra-axial mass bleeding with ventricular rupture in a left fronto-temporal distribution.

The patient is interesting because he is a rare case of MDS with severe thrombocytopenia complicated by spontaneous ICH. ICH was attributed to thrombocytopenia as other causes of bleeding, such as acute hypertensive crisis, coagulation disorder, venous sinus thrombosis with secondary bleeding, ischemic stroke with secondary bleeding, rupture of an aneurysm, drugs, and transformation of MDS to acute myeloid leukemia^4^ were ruled out. There was also no evidence of hemolysis. Thrombocytopenia has previously been identified as a risk factor for ICH^5^. However, low platelet counts do not necessarily lead to bleeding. There have been reports of MDS patients with thromboembolic events despite low thrombocytes.^6^ Thrombotic events in MDS patients with low platelet counts are most likely due to the presence of dysfunctional thrombocytes, which can lead to a susceptibility to thrombus formation. In general, the risk of thrombotic events exceeds the risk of bleeding in patients with MDS.^7^ Although a few patients with MDS and cerebral bleeding have been reported,^3^ spontaneous ICH due to thrombocytopenia is a rare event. With regard to the cause of MDS in the index patient, a comprehensive workup was not carried out due to the short period between the discovery of the pancytopenia and death, the age of the patient, and due to the lack of therapeutic consequences. Although the use of thrombocyte and erythrocyte concentrates showed a favorable effect, it was only temporary. For this reason, the use of azacitidine was considered by the oncologists.

In summary, this case demonstrates that idiopathic MDS can be complicated by spontaneous ICH in the presence of severe thrombocytopenia. Whether pathophysiological mechanisms other than thrombocytopenia contributed to the bleeding, remains speculative and requires further studies.

## Declarations

Informed consent: Was obtained.

Ethics approval: The study was approved by the institutional review board.

Data availability: Not applicable.

Consent to participate: Not applicable.

Consent for publication: Not applicable.

## Author contributions

JF: design, literature search, discussion, first draft, critical comments.

## Conflicts of Interest

The authors declare no conflicts of interest.
